# Electrospray Ionization Tandem Mass Spectrometric Study of Selected Phosphine-Based Ligands for Catalytically Active Organometallics

**DOI:** 10.1021/jasms.3c00104

**Published:** 2023-07-03

**Authors:** Sarah Fleissner, Ernst Pittenauer, Karl Kirchner

**Affiliations:** † Institute of Applied Synthetic Chemistry, TU Wien, Getreidemarkt 9, 1060 Vienna, Austria; ‡ Institute of Chemical Technologies and Analytics, TU Wien, Getreidemarkt 9, 1060 Vienna, Austria

## Abstract

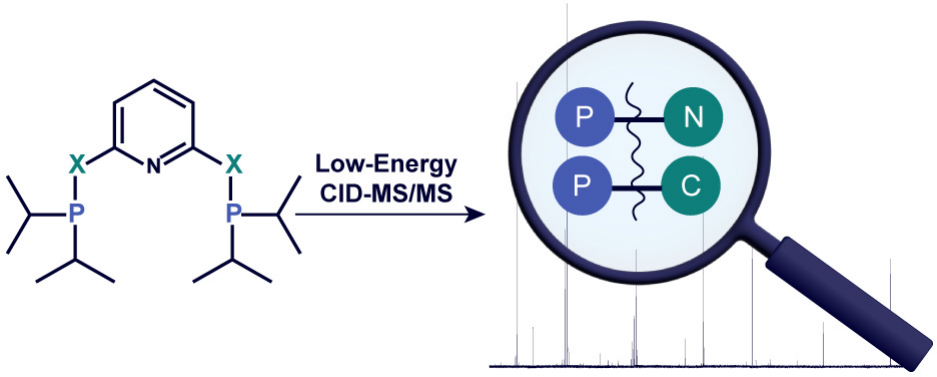

Selected organometallic compounds are nowadays extensively used as highly efficient catalysts in organic synthesis. A great variety of different ligand systems exists, of which phosphine-based ligands are a significant subgroup. While mass spectrometry, predominantly electrospray ionization mass spectrometry (ESI-MS), is a standard analytical technique for the identification of new ligands and their metal complexes, there is little information on the behavior of phosphine-based ligands/molecules by electrospray ionization collision-induced dissociation tandem mass spectrometry (ESI-CID-MS/MS) at low collision energies (<100 eV) in the literature. Here, we report a study on the identification of typical product ions occurring in tandem mass spectra of selected phosphine-based ligand systems by ESI-CID-MS/MS. The influence on the fragmentation behavior of different backbones (pyridine, benzene, triazine) as well as different spacer groups (amine, methylamine, methylene), which are directly linked to the phosphine moiety, is investigated by tandem mass spectrometry. In addition, possible fragmentation pathways are elaborated based on the assigned masses in the tandem mass spectra with high-resolution accurate mass determination. This knowledge may be particularly useful in the future for the elucidation of fragmentation pathways for coordination compounds by MS/MS, where the studied compounds serve as building blocks.

## Introduction

In the field of organometallic chemistry, phosphine-based ligand systems have been proven to be suitable building blocks for the development of effective catalysts for decades.^1^ Mass spectrometry is primarily used as a standard analytical technique in this research area, where the focus is predominantly on the determination of the molecular weight by accurate mass.^2−5^ Despite this, there is very little information in the scientific literature on the fragmentation behavior of phosphine-based ligands or organometallic compounds by tandem mass spectrometry. In the field of biological and organic mass spectrometry, tandem mass spectrometry has been an essential tool for the identification of analytes for many years.^6−8^ In addition, numerous fragmentation mechanisms are well investigated and described.^9−15^ Yet, this knowledge is difficult to apply when working with phosphine-based ligands or even organometallic compounds. In a detailed study of small organic compounds of group V elements carried out by Kostyanovski et al., phosphines were investigated by electron ionization (EI) mass spectrometry.^16,17^ The fragmentation behavior of phosphine-based ligands as well as organometallic compounds utilizing electrospray ionization (ESI), which is widely used today, has not yet been studied more extensively.^18−20^ However, the information on this might be useful when it comes to investigating reactions or even reaction intermediates for example in synthesis and catalysis by means of mass spectrometry and thus being able to assign the structure of the mass values detected.^21^ In the following, we report on the fragmentation behavior of selected phosphine-based ligands utilizing electrospray ionization in combination with collision-induced dissociation tandem mass spectrometry (ESI-CID-MS/MS) on a quadrupole/reflectron time-of-flight (QTOF) mass spectrometer. The emphasis was focused on differences between the compounds in terms of backbone (pyridine, benzene, triazine) and spacer groups (amine, methylamine, methylene) linked to a phosphine moiety, regarding their fragmentation behavior. Special interest is also given to the behavior of P–N bonds under CID, since they are often part of phosphine-based ligand systems. In addition, possible fragmentation pathways are discussed, and parallels are drawn with respect to bond cleavages and rearrangements, as they also occur in small organic molecules that have already been studied extensively by electron ionization mass spectrometry (EI-MS) in the past.

## Experimental Section

### Materials

All samples investigated were synthesized according to the literature by our research group.^22−28^ The analytes were dissolved in methanol just prior to measurement, resulting in a final concentration of 0.1 mg/mL. Methanol (≥99.9%) was obtained from Honeywell (Morristown, NJ, USA). The introduction of the sample into the ion source of the mass spectrometer was performed via direct infusion utilizing a syringe pump model 100 (KD Scientific, Holliston, MA, USA).

### Instrumentation

Measurements were carried out on an Agilent 6545 QTOF mass spectrometer (Agilent Technologies, Santa Clara, CA, USA). The instrument is equipped with a dual electrospray ion source, a quadrupole, an rf-only quadrupole collision cell, and a dual stage reflectron time-of-flight mass analyzer. The collision cell is operated with nitrogen as collision gas. The collision energy ranged from 22 to 32 eV and was adapted for each sample individually. The calibration was performed utilizing the commercially available ES tuning mix from Agilent Technologies (Waldbronn, Germany). All mass spectra were acquired in positive ion mode with an ion source temperature of 200 °C to avoid thermal degradation of the analytes. Tandem mass spectrometric experiments were conducted with two different isolation widths of the precursor ion – narrow (1.3 *m*/*z*) and medium (4 *m*/*z*). The discussion and interpretation of the data in this work was performed using narrow-mode tandem mass spectra to allow better identification of fragmentation and to avoid isotopic overlap. All recorded high-resolution accurate mass spectra were acquired with a resolution of *R* ≈ 20 000, with a mass deviation generally less than 1 ppm. Data processing was performed with MassHunter Workstation Qualitative Analysis 10.0 software (Agilent Technologies, Santa Clara, CA, USA).

## Results and Discussion

**Scheme 1:**
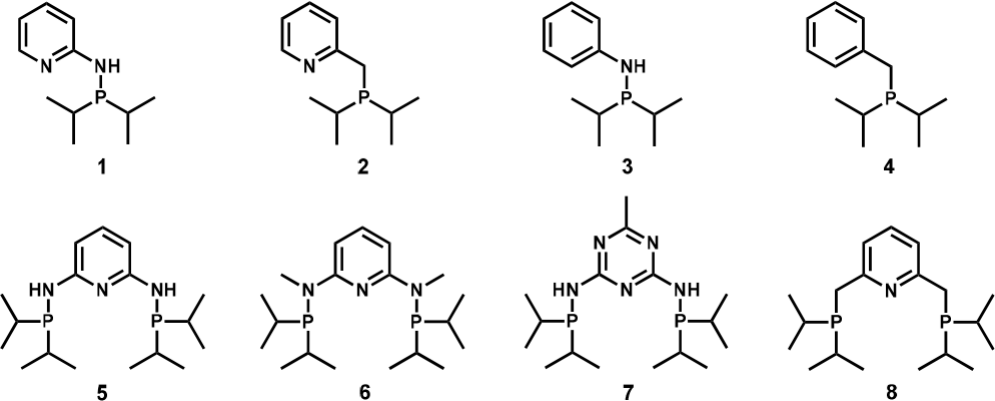
Investigated Phosphine-Based Ligand Systems

In order to gain insight into the fragmentation behavior of phosphine-based ligands and in future their metal complexes by CID- MS/MS, a representative selection of different mono-, bi-, as well as tridentate compounds was investigated (Scheme 1).^29^

### ESI-CID-MS/MS of Mono-/Bidentate Ligand Systems

**Figure 1:**
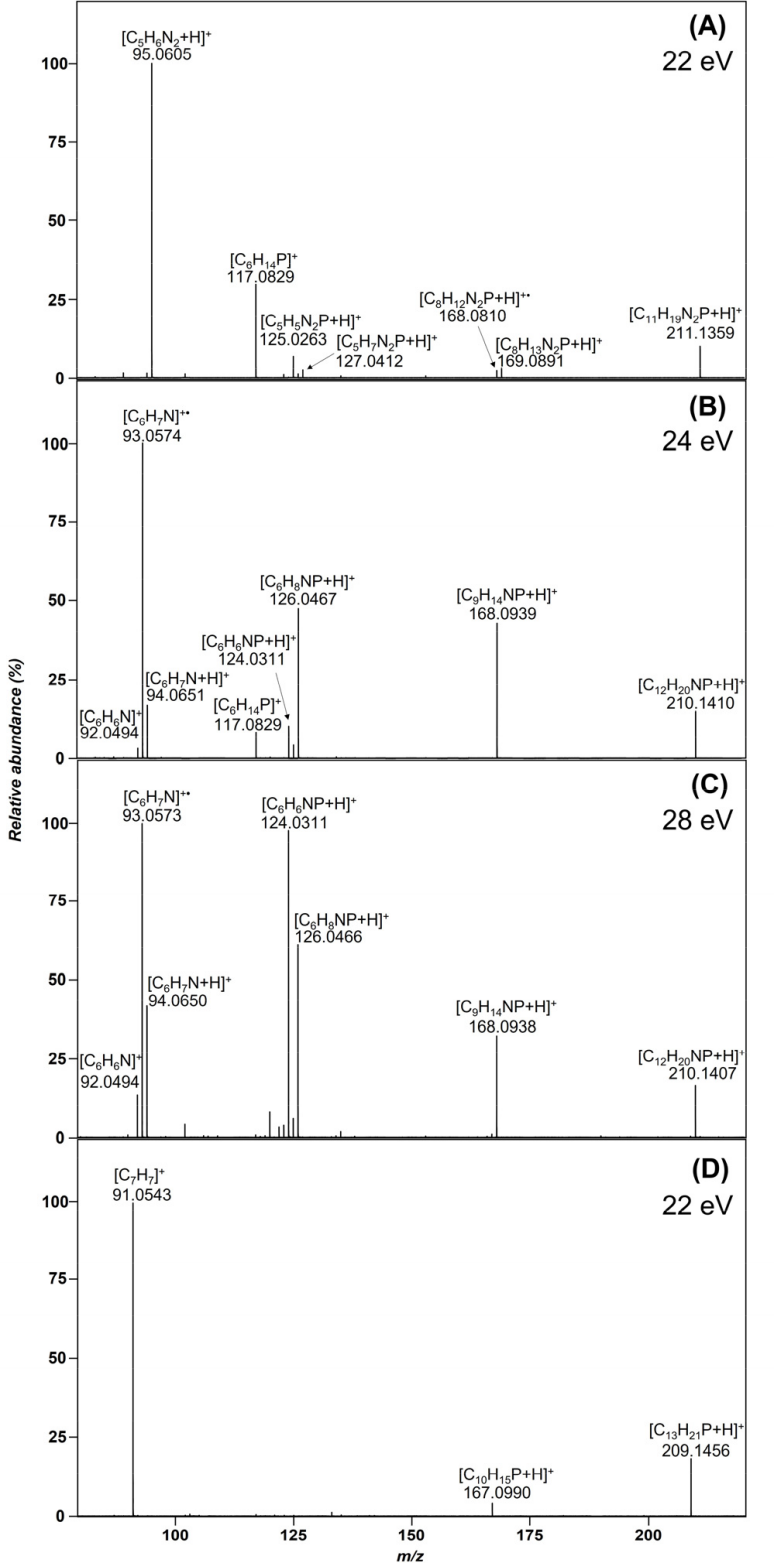
Tandem mass spectra of the mono-/bidentate ligand systems utilizing the [M + H]^+^ molecular ion as a precursor ion (A) of compound **1**, (B) **2**, (C) **3**, and (D) **4**, respectively.

**Scheme 2:**
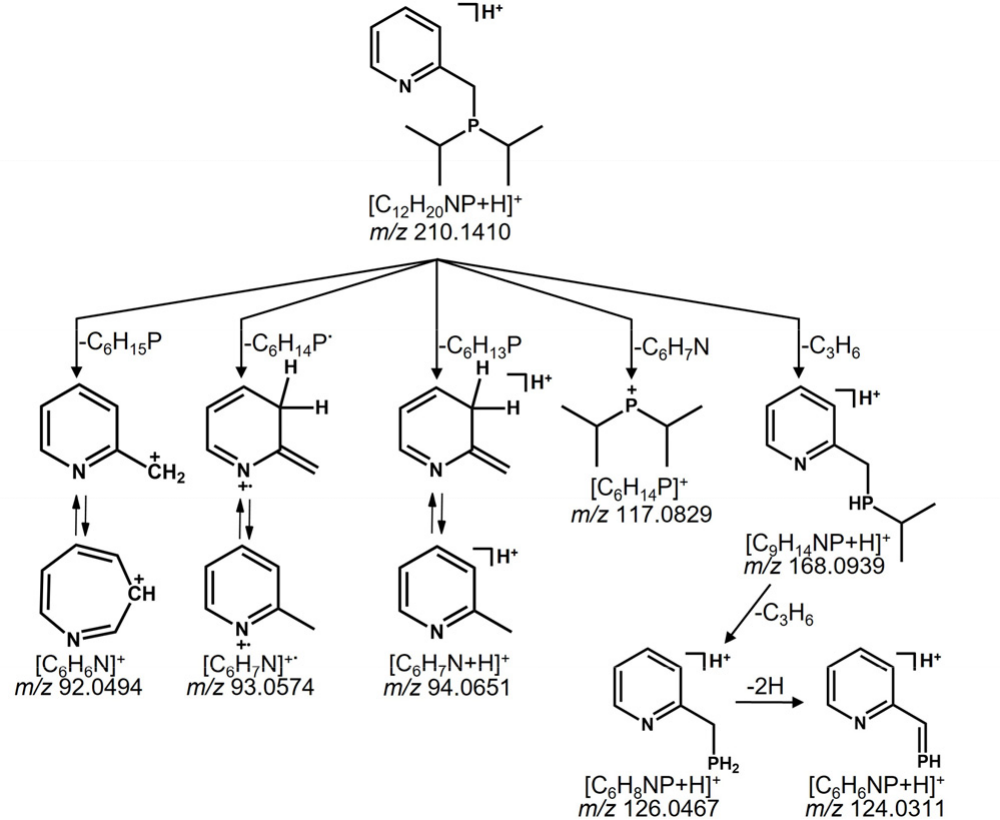
Proposed Fragmentation Pathways of **2** Representative for Mono-/Bidentate Ligand Systems

Initially, ligand systems with one phosphorus donor (compounds **1**–**4**) for potential metal salt complexation were examined. These molecules feature either a pyridine or benzene backbone linked to the phosphine moiety via an amine or methylene group. By varying these structural moieties, the influence of each individual building block of the ligand system on the fragmentation behavior in MS/MS experiments is investigated. Tandem mass spectra of the examined ligands (**1**–**4**) are comparatively depicted in Figure 1. The precursor ion for all compounds is the singly positively charged [M + H]^+^ ion in the corresponding ESI spectrum. Examining the CID spectra more closely, it can be observed that the main fragmentation pathways are basically similar for almost all compounds – only the benzene-based ligand (**4**) stands out at first glance with only one but very dominant fragmentation route that precludes other fragmentation routes, leading to a product ion at *m*/*z* 91.0543. The first fragmentation step for all compounds is the neutral loss of a substituent on the phosphorus atom (loss of propene). Compounds **1**–**3** also exhibit a bond cleavage of the second isopropyl substituent of the phosphorus (loss of either a second propene or loss of a propyl radical plus H spaced by 2 Da). A proposed fragmentation pathway is exemplarily shown for compound **2** in Scheme 2. The final cleavage of the P–X bond (X = CH_2_ or NH) yields quite different types of product ions, depending on the substructure of the aromatic ring system. Whereas compound **1** (*m*/*z* 95.0605) exclusively yields a protonated 2-aminopyridine, compound **4** exhibits a benzyl-/tropylium cation (*m*/*z* 91.0543). As the product ion at a *m*/*z* 95.0605 is obviously formed via a McLafferty-type rearrangement in compound **1**, the product ion at *m*/*z* 91.0543 seems simply to originate from a classical α-cleavage-like fragmentation (benzyl cleavage). In contrast, compounds **2** and **3** exhibit either a 2-methylpyridine or a phenylamine moiety, thus yielding a group of three different product ions of isomeric structure, respectively, for the P–X cleavage. A McLafferty-type rearrangement leads to the product ions at *m*/*z* 94.0650 (protonated 2-methyl-pyridine and protonated aniline). The base peak for compound **2** as well as **3** is represented by a simple homolytic cleavage between the P–N and P–C bonds, respectively, leading to the *m*/*z* 93.0573 product ions, this time representing radical cations. Only a minor fragmentation pathway yields product ions at *m*/*z* 92.0494 formed via an α-cleavage-like fragmentation resulting finally in an aza-tropylium cation. In the spectra of the pyridine-based compounds (Figure 1A,B), the diisopropylphosphine cation is detected as a product ion with different intensities observed at *m*/*z* 117.0829 (**1** and **2**). It is assumed that the cleavage of the P–X bond occurs independently of the backbone (pyridine or benzene), albeit with different intensity. Of notice is the extremely low abundance or absence of the product ion in the benzene-based compounds (Figure 1C,D). Compounds **2** and **3** (see Figure 1B,C) are structural isomers, and it is of particular interest to be able to identify them based on differences in the fragmentation pattern. The product ion with a postulated P=N bond (*m*/*z* 124.0311) shows a much higher abundance in the spectrum than the P=C bond. Moreover, the protonated product ion (*m*/*z* 126.0467) dominates in the CID spectrum of compound **2**. Concerning the origin of the *m*/*z* 126 and 124 product ions, the major fragmentation pathways appear to be consecutive loss of two propene neutrals (*m*/*z* 168 and 126) followed by two protons (*m*/*z* 124) rather than loss of one propene neutral plus an isopropyl radical plus one proton. This observation is strongly supported by a very low-abundant *m*/*z* 125 product ion, thus indicating an isopropyl radical loss as second step.

Furthermore, the diisopropylphosphine cation is present in higher intensity only for compound **2**. These significant differences allow the two structural isomers to be distinguished from each other by ESI-CID-MS/MS.

### ESI-CID-MS/MS of Tridentate Ligand Systems

**Figure 2:**
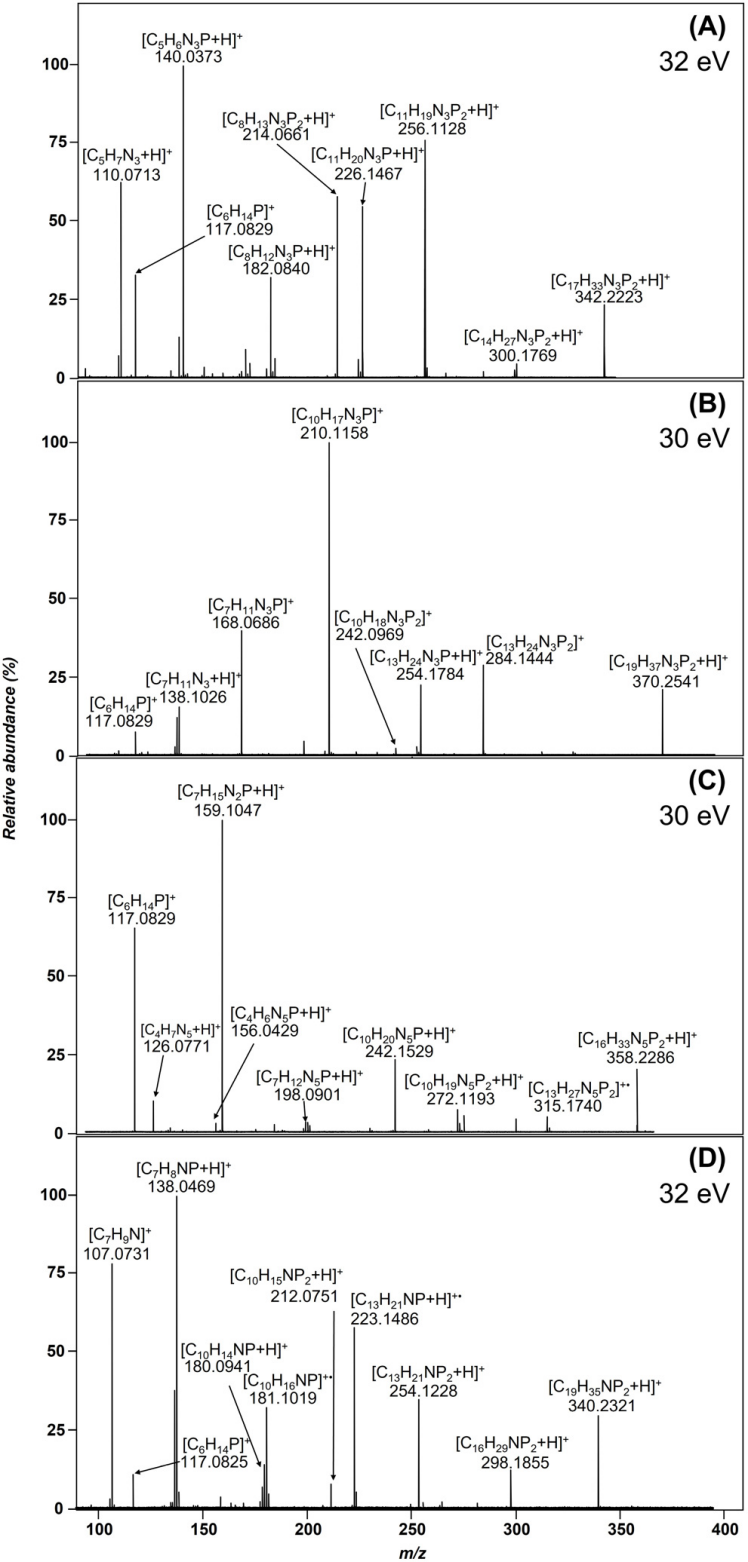
Tandem mass spectra of the tridentate ligand systems utilizing the [M + H]^+^ molecular ion as a precursor ion (A) of compound **5**, (B) **6**, (C) **7**, and (D) **8**, respectively.

**Scheme 3:**
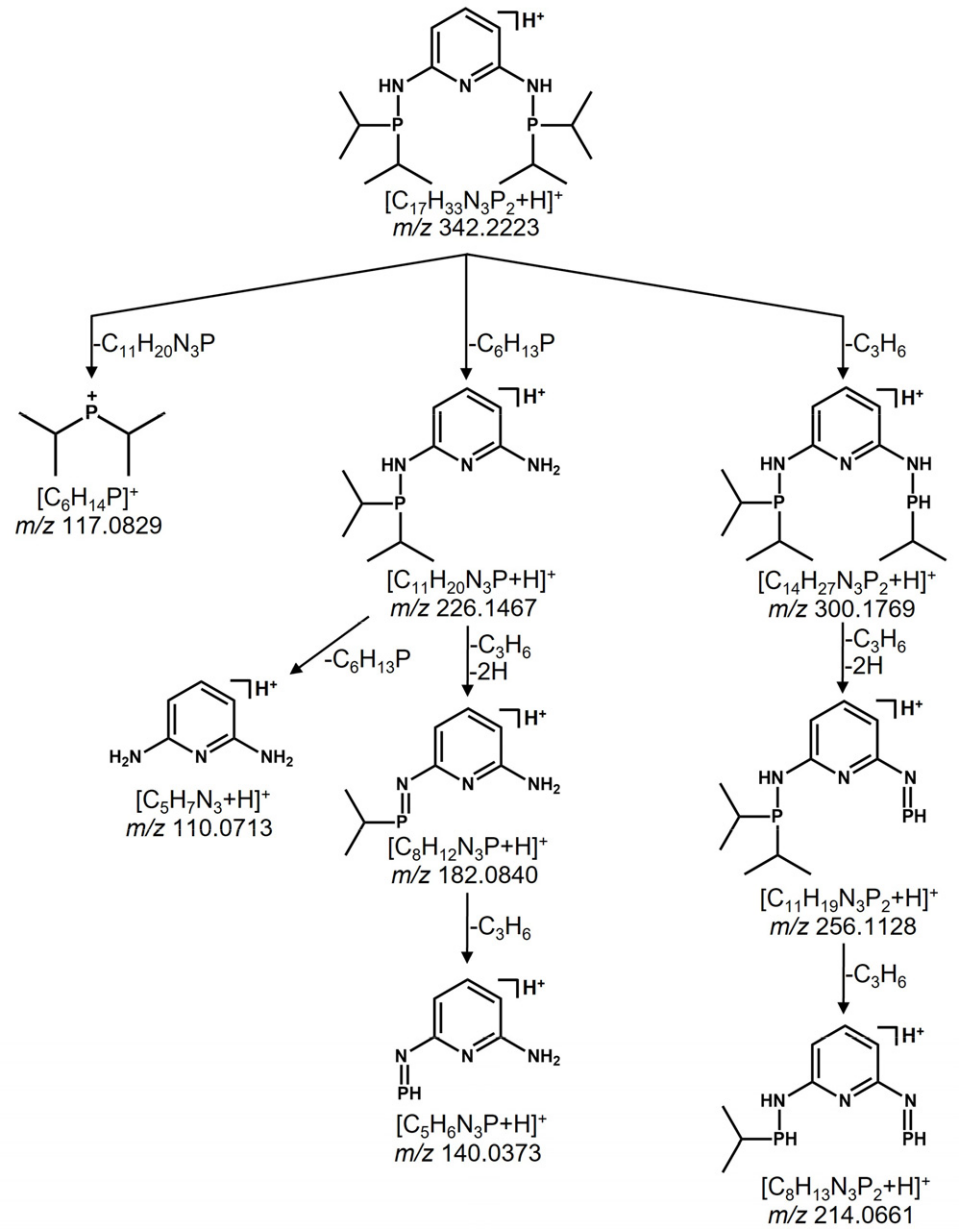
Proposed Fragmentation Pathways of **5**

Following the examination of simple ligands with one phosphine group and evaluating the tandem mass spectra, the tridentate analytes with two phosphine moieties (compounds **5**–**8**) are investigated, and the data are interpreted based on previous findings. These compounds feature a pyridine or triazine backbone, respectively, with either an amine, methylamine, or methylene spacer. Again, the protonated molecule [M + H]^+^ is selected as the precursor ion. The corresponding tandem mass spectra are depicted in Figure 2. Comparing the main fragmentation pathways of all tridentate compounds, they behave quite similar at first glance under CID-MS/MS conditions, except for compound **7**, which shows a rather low-abundant fragmentation with only two prominent low-mass product ions, compared to **5**, **6**, and **8**. Table 1 compares neutral losses of the investigated compounds **5**–**8**, and Scheme 3 shows a proposed fragmentation pathway of compound **5** representative for the examined tridentate systems. The cleavage of a propene group of the phosphorus substituent is indicated through a neutral loss of 42 Da, occurring in sample **5** (*m*/*z* 300.1769) and **8** (*m*/*z* 298.1855). An additional fragmentation resulting in a loss of a propyl radical (loss of 43 Da) is observed exclusively in Figure 2C of compound **7**. No product ion with a mass difference of either 42 or 43 Da can be assigned to a signal in the tandem mass spectrum of compound **6**. A neutral loss of 86 Da indicates loss of propene and a propyl radical plus hydrogen or, alternatively, two propyl radicals respectively. This type of product ion is detected in all compounds investigated. It cannot be determined if the bond cleavage of the two substituents occurs at the same phosphorus atom or one substituent of each phosphorus atom. Since in the compounds with one phosphine moiety a cleavage of both substituents takes place, it is reasonable to assume that this behavior is found in samples with two phosphines. In the case of compound **6**, which is the only compound that cannot contain a protonated product ion by a loss of 86 Da, it is assumed that the positive charge is now located at the quaternized nitrogen atom of the methylamine spacer. The neutral loss of 116 Da corresponds to the loss of an isopropyl-isopropylidenephosphine neutral, which appears in compounds **5**–**7**, whereas compound **8** shows a loss of 117 indicating an α-cleavage-like fragmentation by loss of a bis(isopropyl)phosphine radical (*m*/*z* 223.1486). A further loss of 128 Da for compound **5** most likely occurs via loss of propene (−42 Da), followed by loss of propene plus two protons and finally again by loss of propene. In contrast, compound **6** immediately shows a loss of 86 Da (here, a distinction between loss of two propene neutrals plus two protons versus loss of two isopropyl radicals cannot be made) followed by loss of propene. Compound **7** only shows this fragmentation by consecutive loss of two isopropyl radicals, but no further fragmentation leading to a [M + H – 128]^+^ fragmentation. Finally, compound **8** shows the identical fragmentation behavior as compound **5**.

**Table 1:** Comparison of Neutral Losses of the Investigated Tridentate Ligands **5**–**8**^a^

	**5**	**6**	**7**	**8**
**[M + H]**^**+**^	342.2223	370.2541	358.2286	340.2321
**Neutral loss**				
42	300.1769	-	-	298.1855
43	-	-	315.1740	-
86	256.1128	284.1444	272.1193	254.1228
116	226.1467	254.1784	242.1529	-
117	-	-	-	223.1486
128	214.0661	242.0969	-	212.0751
160	182.0840	**210.1158**	198.0901	180.0941
202	**140.0373**	168.0686	156.0429	**138.0469**
232	110.0713	138.1026	126.0771	-
*m*/*z* 117	+	+	+	+
**Cross ring fragmentation**	-	-	**159.1047**	-

a Masses in bold mark the base peak.

In contrast, the neutral loss of 160 Da is only detected for compound **6** with significant abundance. The combined loss of −(42 + 44 + 116) Da or alternatively −(43 + 43 + 116) Da resulting in a fragmentation [M + H – 202]^+^ is the base peak for compounds **5** and **8**. For all other compounds, this fragmentation is of minor abundance. The simultaneous loss of two isopropyl-isopropylidene phosphine neutrals (2 × 116 = 232 Da) is detected in the tandem mass spectra of compounds **5**–**7** as a protonated product ion (*m*/*z* 110.0713; *m*/*z* 138.1026; *m*/*z* 126.0771). In contrast, for compound **8**, a radical product ion (*m*/*z* 107.0731) is identified as loss of 233 Da (116 + 117). As mentioned above, this is also the only compound that shows a loss of the diisopropylphosphine radical (117 Da) instead of a loss of an isopropyl-isopropylidenephosphine (116 Da). The diisopropylphosphine cation (*m*/*z* 117.0829) occurs in all spectra of the tridentate compounds (**5**–**8**) studied, although in vastly different intensities. While the highest relative abundance of this cation appears in Figure 2C, for the compound with the triazine backbone (**7**), the lowest intensities are observed in Figure 2B,D, for the pyridine-based compounds with a methylamine (**6**) or methylene (**8**) spacer group, respectively. If these experimental results are now compared with pyridine-based bidentate ligands studied (**1**,**2** vide supra), it can be observed that the diisopropylphosphine cation also appears at a similar intensity in compounds with two phosphine moieties. Furthermore, a trend can be observed here which directly relates the influence of the spacer group to the occurrence of the diisopropylphosphine cation. Basically, the following applies: amines > methylamine∼methylene. Additionally, we observe an unexpected cross ring fragmentation, which takes place exclusively in compound **7** constituting a triazine backbone. A possible explanation is the decreasing basicity due to the increasing number of nitrogen atoms and the increased localization of the π-electrons, which results in a more labile ring system with less aromatic character.^30^

## Conclusion

This study focused on the investigation of the fragmentation behavior of selected phosphine-based compounds using ESI-CID-MS/MS. For the examined ligands with one phosphine moiety, protonated, single cationic and radical cationic product ions resulting from hetero- and homolytic cleavages, respectively, as well as McLafferty-type rearrangements, could be assigned. Two structural isomers (**2** and **3**) have also been distinguished by the different preference of certain fragmentation pathways as well as the presence of the free diisopropylphosphine cation as product ion (visible in most pyridine-based systems – absent in benzene-based compounds). In the investigation of tridentate ligands (two phosphine moieties), the fragmentation behavior shows great similarities to the bidentate samples. The exception to the rule is the fragmentation pattern of the triazine compound, where, in addition to fragmentation with low abundance, cross-ring fragmentation on the triazine ring occurs and can be assigned to the base peak in the CID-spectrum. No such fragmentation was observed for the analytes containing a pyridine or benzene moiety. These results contribute to a toolbox for further investigation of organometallic compounds containing phosphine moieties by tandem mass spectrometry and, particularly, for the identification of these compounds in reaction solutions, where a complex sample matrix predominates.

## Supporting Information Available

Tandem mass spectra of mono-/bi- and tridentate ligands recorded with a medium (4 *m*/*z*) isolation width of the precursor ion (PDF)
