# Supplementary material for: Electrospray Ionization Tandem Mass Spectrometric Study of Selected Phosphine-Based Ligands for Catalytically Active Organometallics
Source: J Am Soc Mass Spectrom. 2023 Jul 3;34(8):1647–52. doi: 10.1021/jasms.3c00104 (PMC10401707; doi:10.1021/jasms.3c00104)
Supplement: Supplementary file 1 — js3c00104_si_001.pdf [file js3c00104_si_001.pdf]

Supporting Information

Electrospray ionization tandem mass spectrometric study of selected phosphine-based ligands for catalytically active organometallics

Sarah Fleissner[1], Ernst Pittenauer[2]* and Karl Kirchner[1]*

[1]Institute of Applied Synthetic Chemistry, TU Wien, Getreidemarkt 9, 1060 Vienna, Austria
[2]Institute of Chemical Technologies and Analytics, TU Wien, Getreidemarkt 9, 1060 Vienna, Austria

Corresponding author: Ernst Pittenauer; Email: ernst.pittenauer@tuwien.ac.at
Karl Kirchner; Email: karl.kirchner@tuwien.ac.at

Contents

1. Tandem mass spectra of mono-/bidentate ligands recorded with a medium (4 m/z) isolation width of the precursor ion.

2. Tandem mass spectra of tridentate ligands recorded with a medium (4 m/z) isolation width of the precursor ion.

1. Tandem mass spectra of mono-/bidentate ligands recorded with a medium (4 m/z) isolation width of the precursor ion.

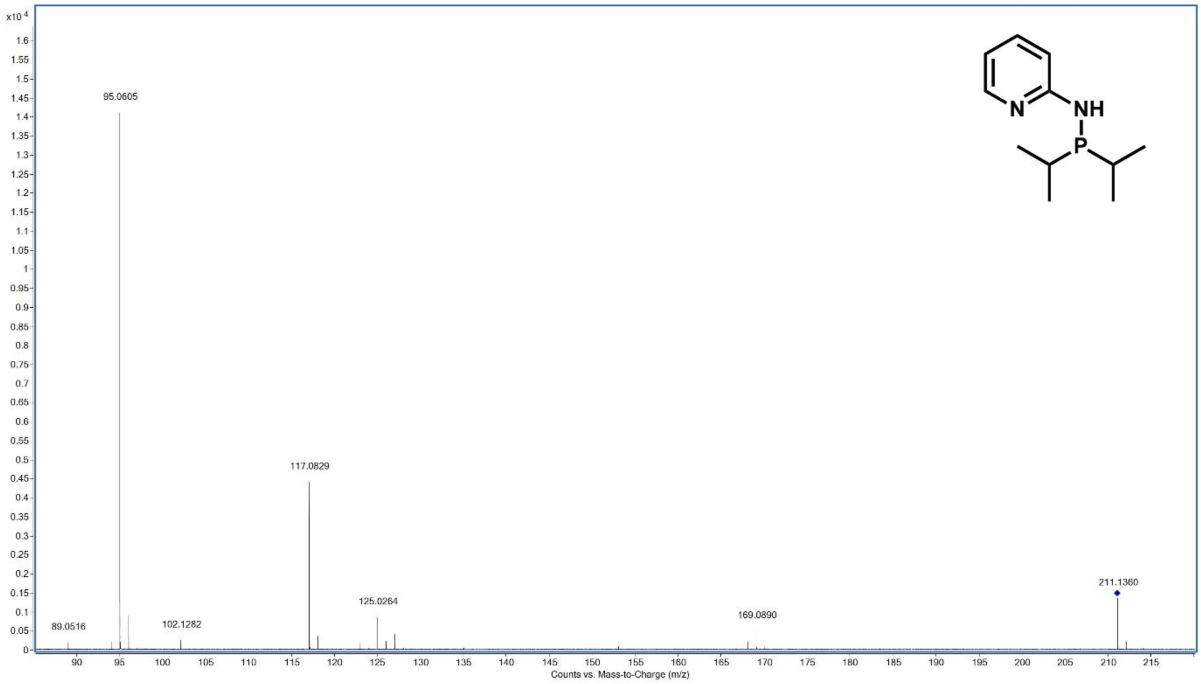

Figure S1. Tandem mass spectrum of **1** with a medium isolation width (4 m/z) of the precursor ion $[M+H]^+$.

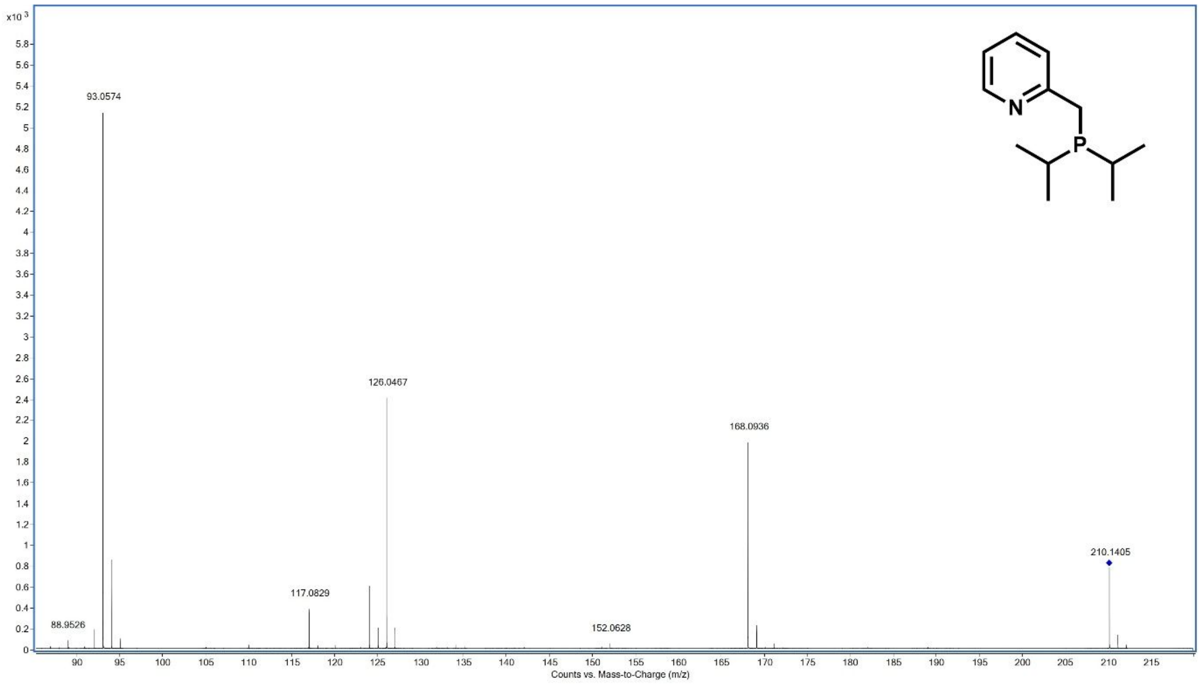

Figure S2. Tandem mass spectrum of **2** with a medium isolation width (4 m/z) of the precursor ion $[M+H]^+$.

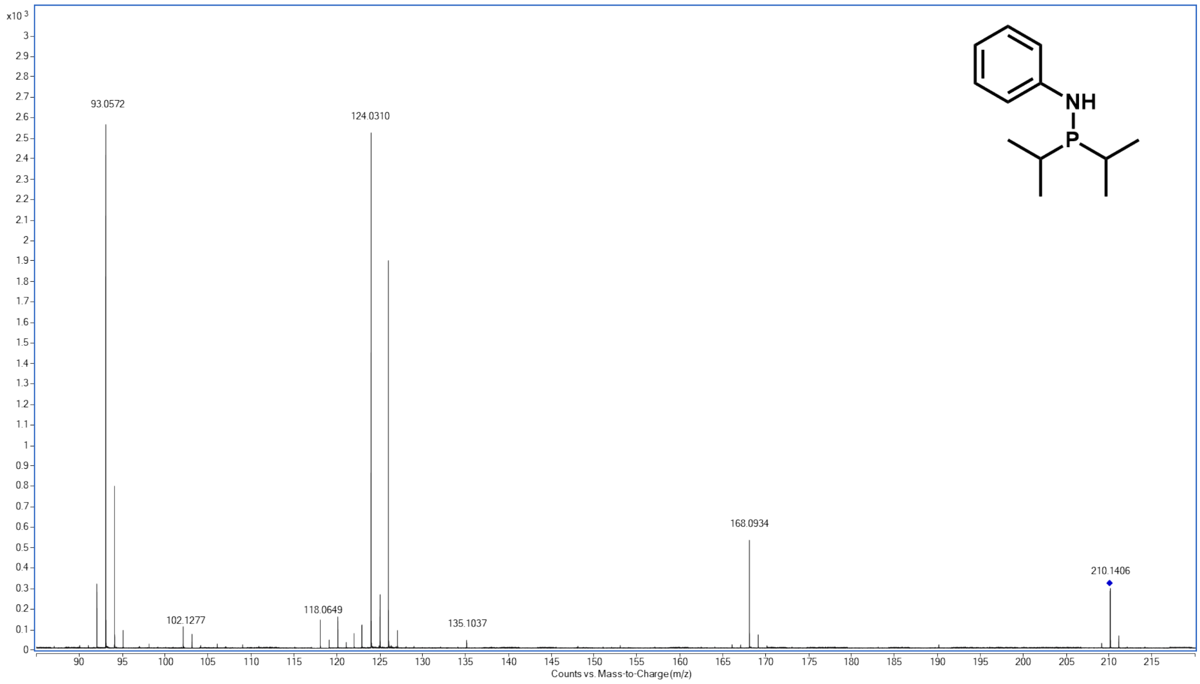

Figure S3. Tandem mass spectrum of **3** with a medium isolation width (4 m/z) of the precursor ion $[M+H]^+$.

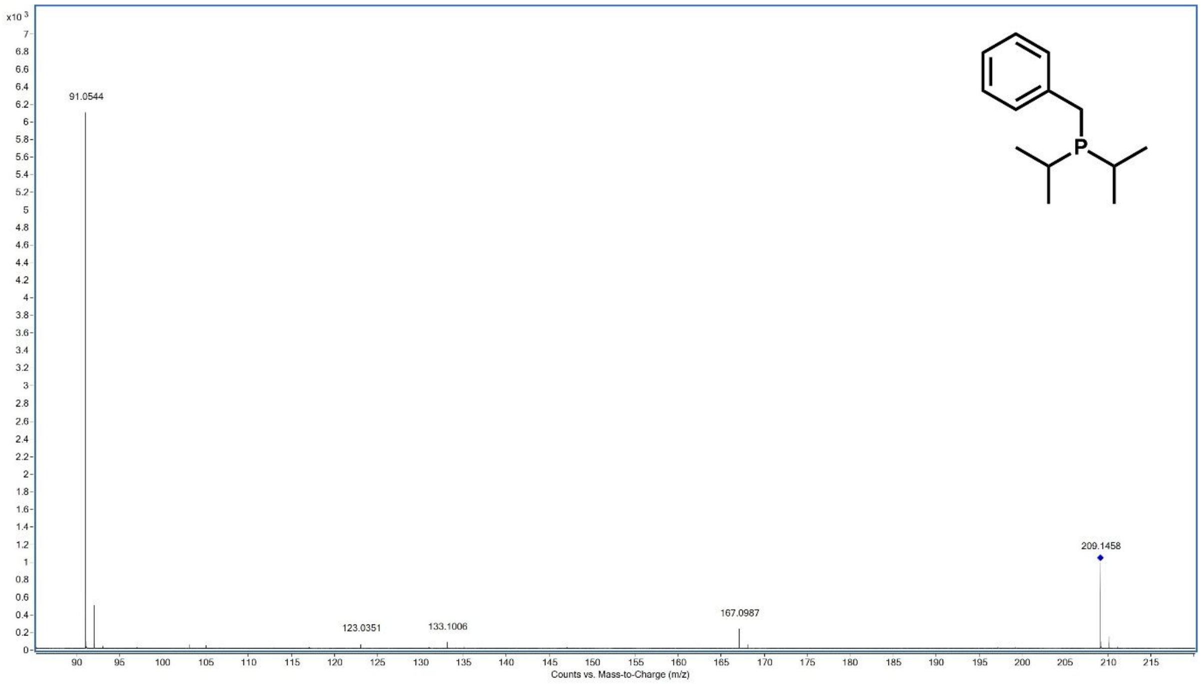

Figure S4. Tandem mass spectrum of **4** with a medium isolation width (4 m/z) of the precursor ion $[M+H]^+$.

2. Tandem mass spectra of tridentate ligands recorded with a medium (4 m/z) isolation width of the precursor ion.

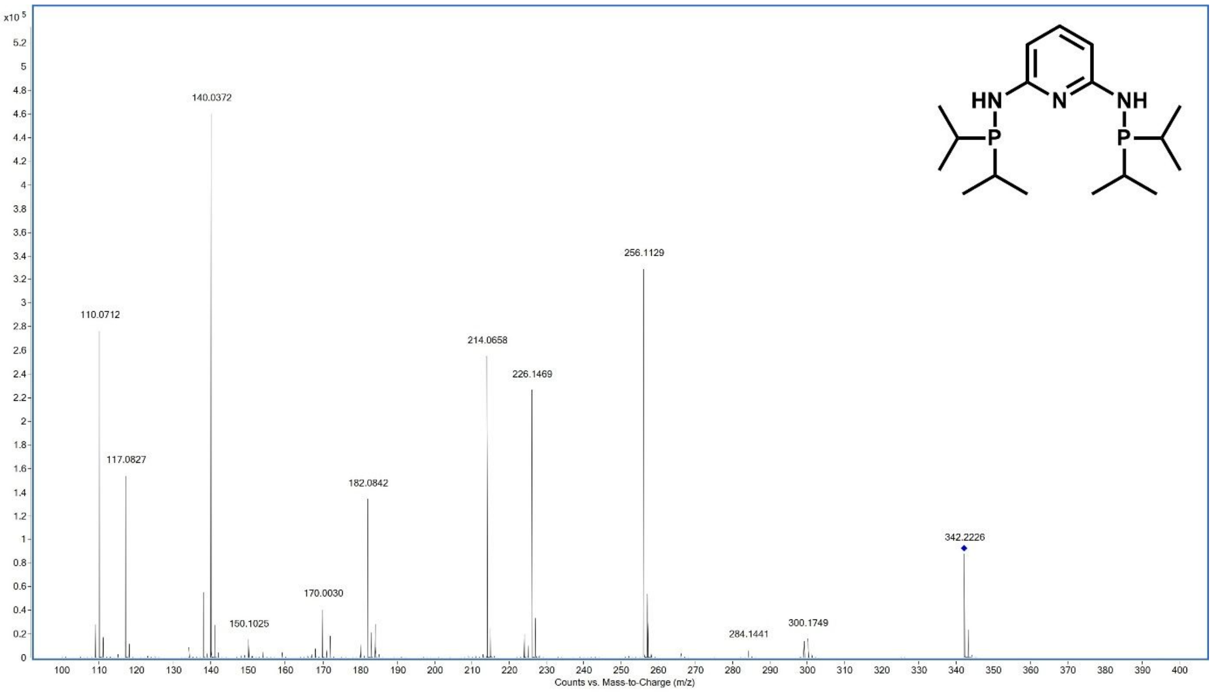

Figure S5. Tandem mass spectrum of **5** with a medium isolation width (4 m/z) of the precursor ion $[M+H]^+$.

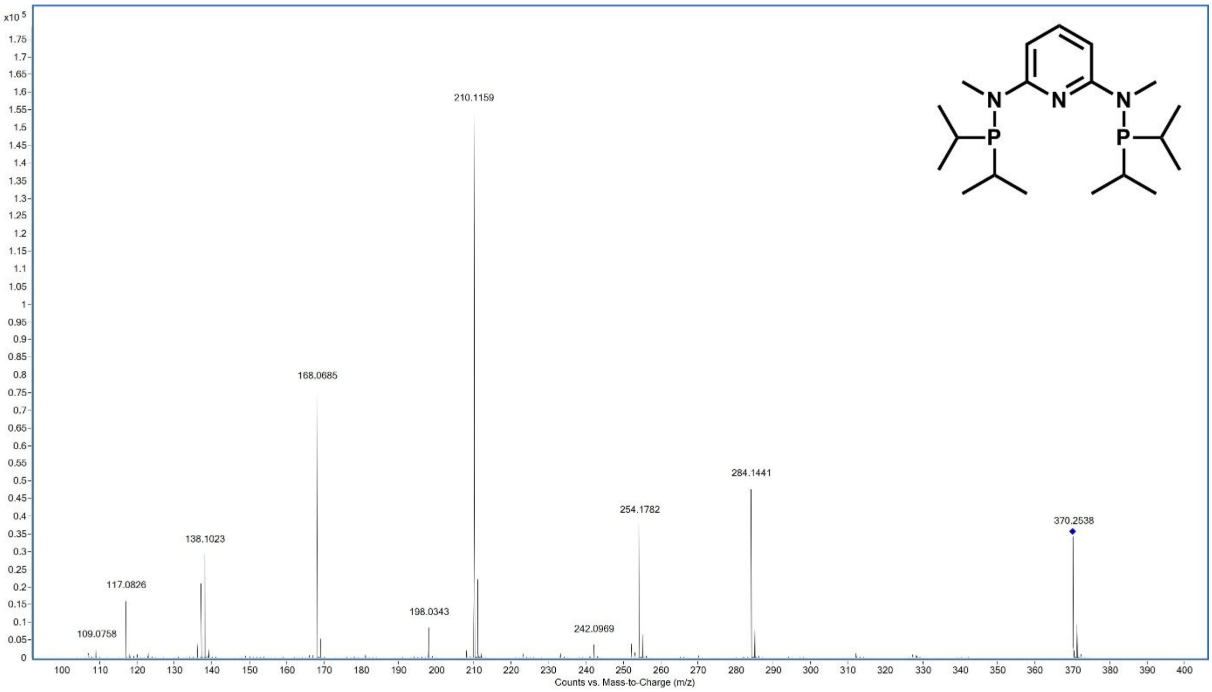

Figure S6. Tandem mass spectrum of **6** with a medium isolation width (4 m/z) of the precursor ion $[M+H]^+$.

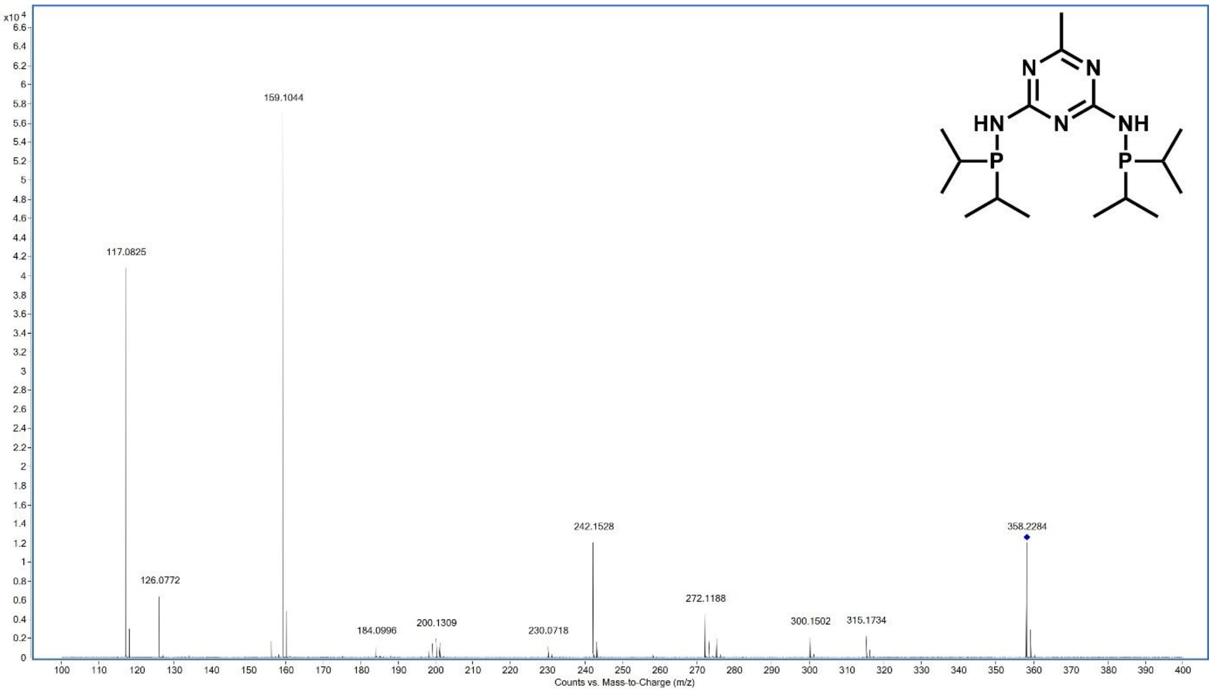

Figure S7. Tandem mass spectrum of **7** with a medium isolation width (4 m/z) of the precursor ion $[M+H]^+$.

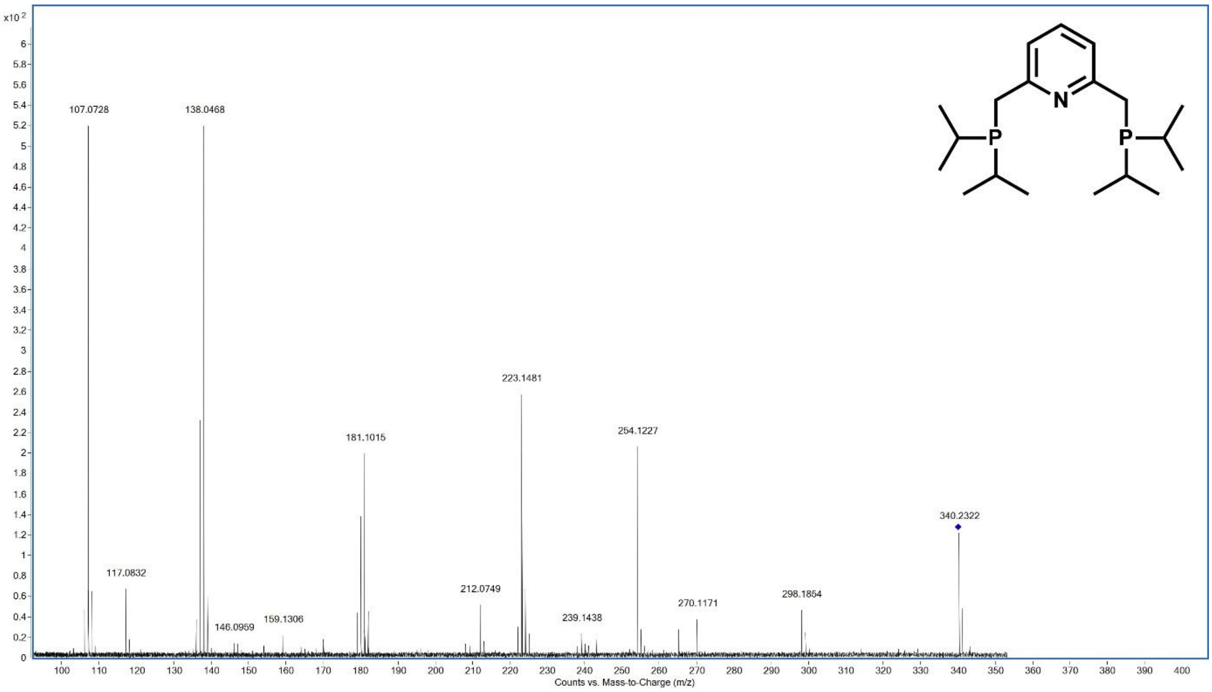

Figure S8. Tandem mass spectrum of **8** with a medium isolation width (4 m/z) of the precursor ion $[M+H]^+$.
